# An Enzymatic Route to Selenazolines

**DOI:** 10.1002/cbic.201300037

**Published:** 2013-02-18

**Authors:** Jesko Koehnke, Falk Morawitz, Andrew F Bent, Wael E Houssen, Sally L Shirran, Matthew A Fuszard, Iain A Smellie, Catherine H Botting, Margaret C M Smith, Marcel Jaspars, James H Naismith

**Affiliations:** [a] Biomedical Science Research Complex, University of St AndrewsNorth Haugh, St. Andrews, KY16 9ST (UK); [b] Institute of Medical Sciences, University of AberdeenAshgrove Road West, Aberdeen, AB25 2ZD (UK); [c] Marine Biodiscovery Centre, Department of Chemistry, University of AberdeenMeston Walk, Aberdeen, AB24 3UE (UK)

**Keywords:** biochemistry, biosynthesis, patellamides, selenazolines

Selenium heterocycles (e.g., selenazofurin) have a number of potential applications in medicinal chemistry; although isosteric with sulfur and oxygen, they have different chemical properties. As a route to exploring biological properties, this substitution is a valuable addition to the chemist's arsenal. The chemical synthesis of selenazolines and selenazoles involves the use of stoichiometric quantities of toxic selenium-containing precursors. However, we have incorporated selenocysteine into a substrate ribosomal peptide by feeding dilute solutions of selenocysteine to *E. coli*. We demonstrate that the dehydrating heterocyclases from the patellamide and the closely related trunkamide biosynthetic pathways process selenocysteine-containing substrate to yield selenazoline, thus establishing an enzymatic route to selenazolines. Such groups can also be incorporated into macrocyclic peptides. Although this remains unknown, it is possible that nature itself could utilise the same enzymes to produce selenium variants of natural products.

1,3-Selenazoles are intriguing structural motifs in medicinal chemistry^1^ as they serve as analogues of the corresponding thiazoles; arguably the best-known examples are the synthetic compounds selenazofurin^2^ (**1**) and amselamine^3^ (**2**; Scheme 1). Zhang et al.^4^ recently reported the chemical synthesis of selenazole-containing cyclic peptides (e.g., **3**) and their application in X-ray diffraction studies of P-glycoprotein. 1,3-Selenazolines1a have also received attention in recent years,^5^ for example, Withers et al.5a have reported the synthesis of a GlcNAc-derived 1,3-selenazole that was evaluated as a potential *O*-GlcNAcase inhibitor. Most synthetic routes to 1,3-selenazoles are based on the Hantzsch synthesis,1a, 5c, 6 although other methods have been investigated.^7^

**Scheme 1:**
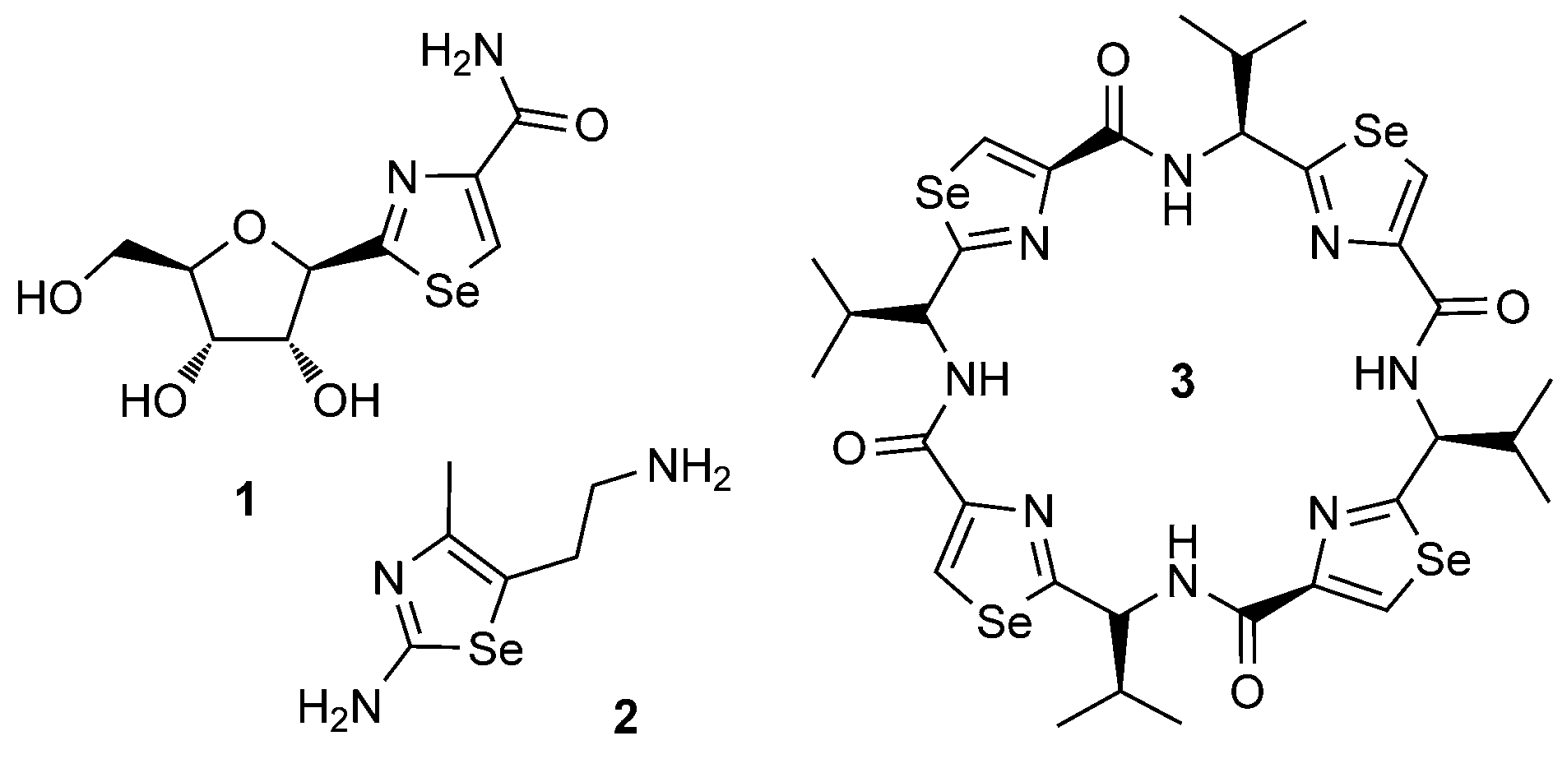
Structures of selenazofurin (**1**), amselamine (**2**) and the chemically synthesised patellamide-like QZ60-SSSS (**3**).

Cyanobactins are a family of ribosomally derived cyclic peptides containing six to 20 amino acids, they are produced from a larger precursor peptide and contain extensive chemical modifications including heterocycles, d-stereocentres and disulfide bonds. They are potent biological scaffolds that can cross cell membranes and are resistant to enzymatic degradation.^8^ Patellamides are members of the cyanobactin family that are produced by *Prochloron didemni*, an obligate symbiont of the sea squirt *Lissoclinum patella* found in Pacific reefs.^9^ The biosynthetic pathway involves the ATP-dependent heterocyclase PatD, protease PatA and oxidase/macrocyclase PatG, which convert the ribosomal precursor peptide PatE into the final macrocycle (Scheme 2). Patellamides and variants can be produced heterologously in *E. coli* by coexpressing a mutant PatE with the biosynthetic genes.^10^

**Scheme 2:**
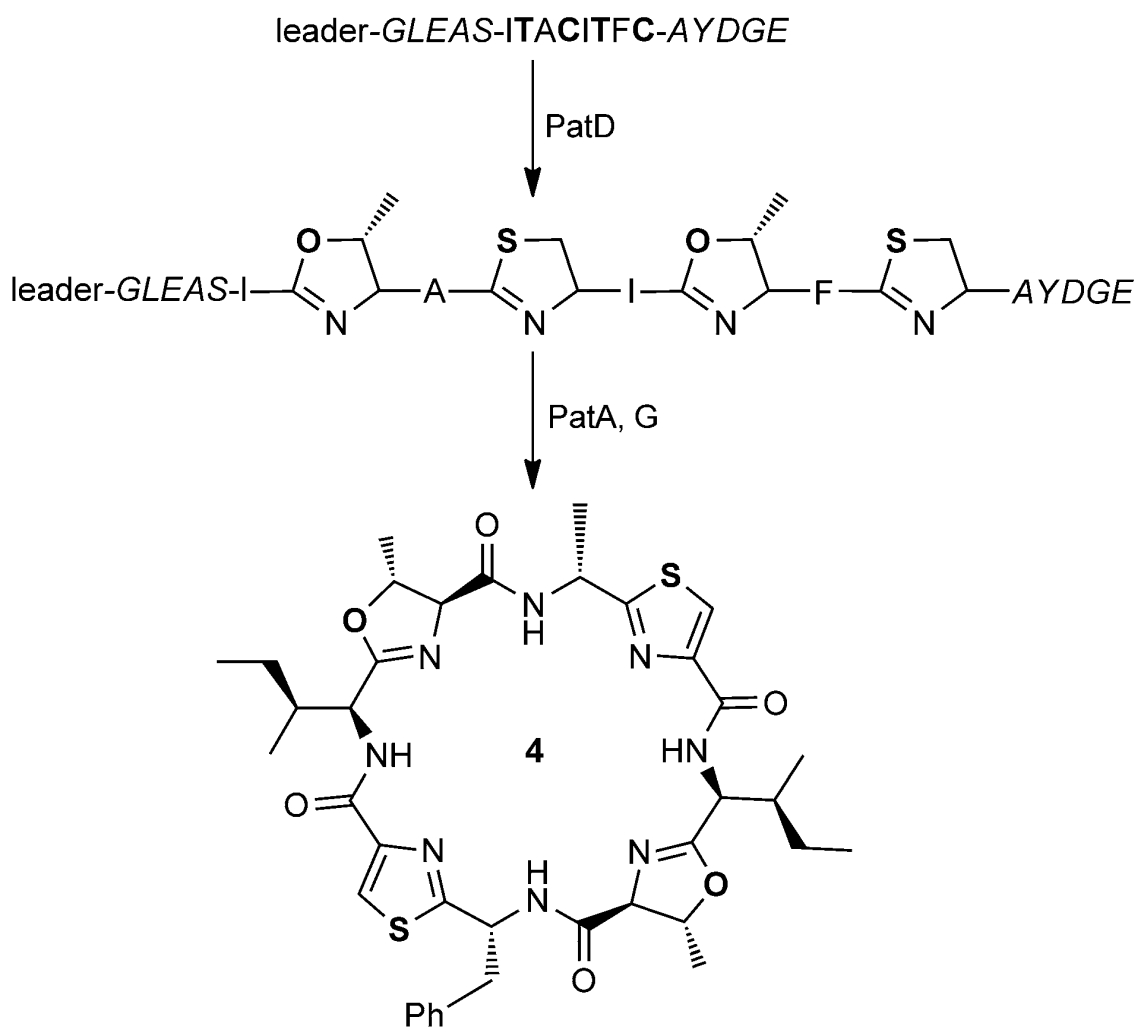
Cassette residues of the precursor peptide PatE are heterocyclised by PatD. The leader peptide is needed for processing but is subsequently cleaved off by PatA. The timing and cause (enzymatic or spontaneous) of epimerisations are not known. PatG oxidises the thiazoline, cleaves off the AYDGE from the C terminus and macrocyclises the eight-residue peptide to give the final product patellamide D (**4**).

Herein we describe the biosynthesis of a selenol-containing PatE from selenocysteine (SeCys). SeCys is commercially available and significantly less toxic than many other selenium precursors. We show that the selenium variant protein is a substrate for PatD (and its homologue from the trunkamide pathway, TruD),^9^, ^10^ which heterocyclises cysteine residues to thiazolines. This enzymatic route thus offers a mild and convenient laboratory method for the synthesis of selenazoline (Sezn) within the context of a peptide.

We purified a modified PatE^9^ precursor peptide that has a single product cassette containing the sequence ITASITFC and in which the cysteine is replaced by SeCys by using the medium supplementation method. We confirmed the incorporation of Se by mass spectrometry and denoted the product PatE(Se). We also observed PatE in which the selenol was hydrolysed to serine (PatE(O), 6828.6 Da, Figure 1); presumably this is a consequence of the growth and protein purification process. Selenol (like thiol) reacts with iodoacetamide (IAA) to give a covalent adduct, as can be seen in Figure 1. We reasoned that addition of IAA to TruD-treated PatE, which has heterocyclic Sezn, would not lead to a reaction, and thus no mass shift would be seen. We saw that PatE that had reacted with TruD (and PatD), and thus lost a water molecule, no longer reacts with IAA (Figure 1); this is consistent with loss of selenol reactivity as the result of formation of a heterocycle.

**Figure 1:**
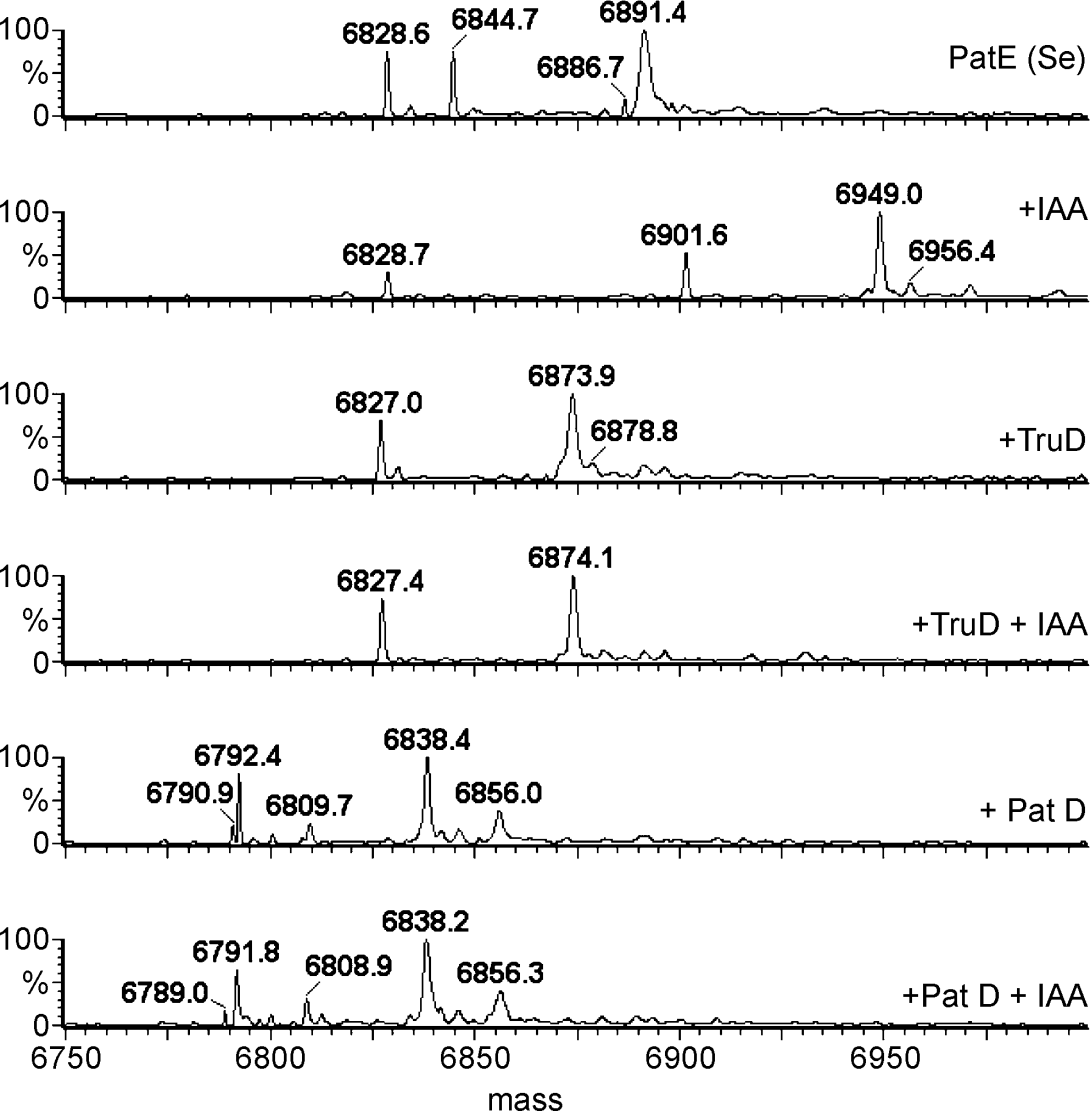
MALDI spectra of PatE(Se), after treatment with iodoacetamide (IAA), incubation with TruD, incubation with TruD followed by IAA, incubation with PatD and incubation with PatD followed by IAA. PatE(Se) shows both Se- (6891.4 to 6949.0 Da) and S- (6844.7 Da to 6901.6 Da) containing compounds. IAA reacts with free selenols (and thiols) adding 57 Da. TruD incubations show loss of 18 Da (one heterocyclisation), PatD loss of 36 and 54 Da (two and three heterocyclisations). After TruD or PatD treatment, IAA has no effect, thus indicating that free selenol/thiol is no longer present in the molecule. Molecules and expected weights are detailed in Table S1.

We digested TruD- and PatD-treated PatE(Se) with the protease GluC, which cleaves after acid residues, to produce smaller molecules for more precise analysis. GluC treatment yielded ASITASITF(Sezn)AYDGELE but did not cleave between C-terminal E and L, possibly because the preceding Sezn heterocycle inhibited proteolysis. For the TruD-treated PatE(Se), we observed a doubly charged fragment of *m*/*z* 910.8847 that has the expected isotope pattern for the incorporation of selenium into the molecule and matches the predicted mass of ASITASITF(Sezn)AYDGELE (+6.5 ppm; Figure 2 A).

**Figure 2:**
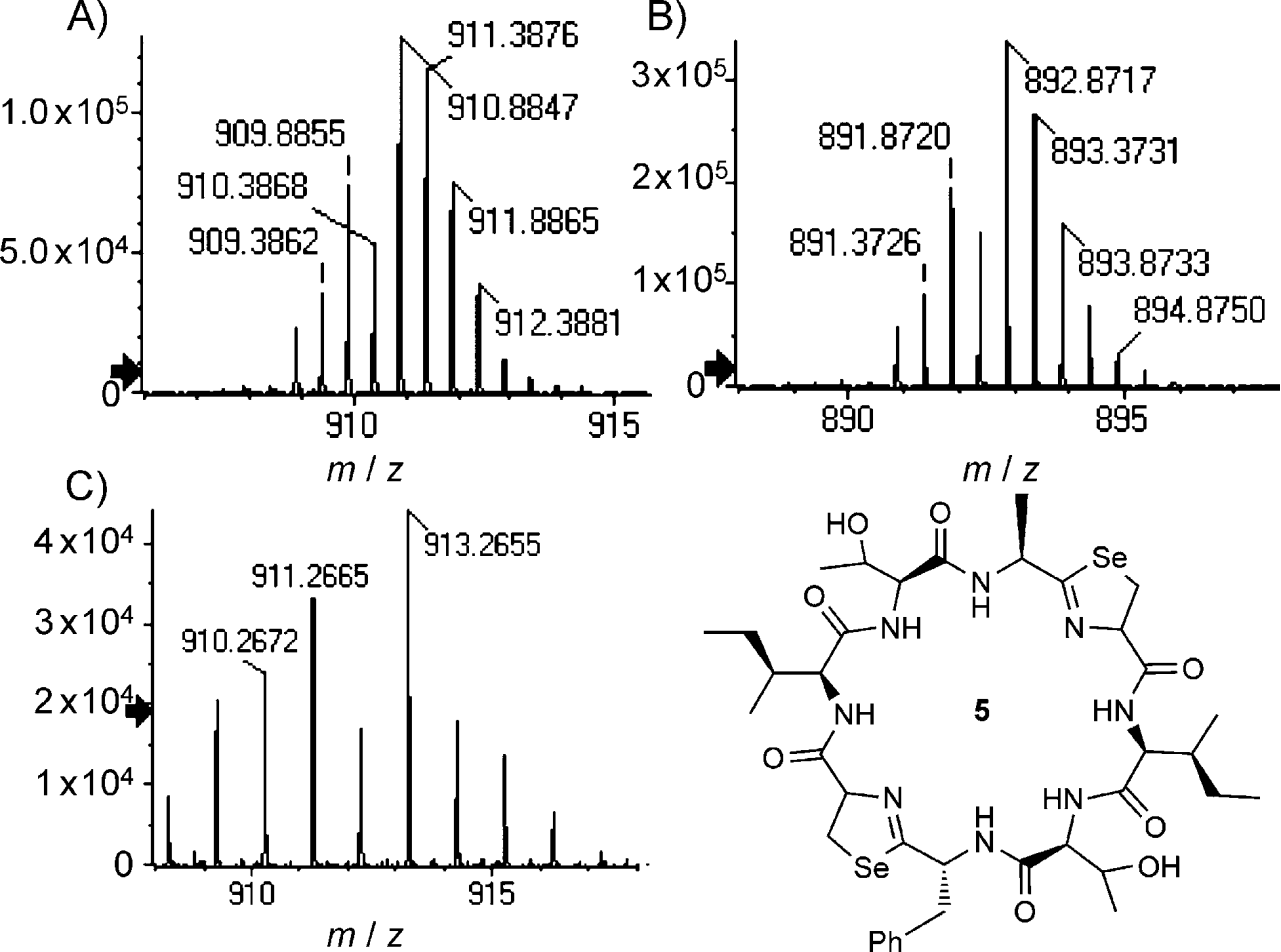
A) Doubly charged ASITASITF(Sezn)AYDGELE, expected *m*/*z* 910.8787 Da (+6.5 ppm) with the characteristic Se isotope pattern. B) Doubly charged ASITF*S*IT*F(Sezn)AYDGELE with three heterocycles (PatD processes alcohols, we cannot assign which of the alcohols (marked by *) are processed in addition to SeCys, more detail in Table S3), expected *m*/*z* 892.8681 (+4.0 ppm). C) Macrocyclised ITA(Sezn)ITF(Sezn) (**5**), expected *m*/*z* 913.2627 (+3.1 ppm).

For the PatD-processed PatE(Se), we observed a doubly charged fragment of 892.8717 Da with a selenium isotope pattern that matches ASITASITF(SeCys)AYDGELE with three heterocycles (+4.0 ppm; Figure 2 B). Proteolytic fragments were analysed by MS/MS, and the peptide sequences were determined by analysing the internal cleavage, b- and y-ion series (Figure S1 A and B). We were able to sequence the peptides to within one residue of the expected Sezn on both the N- and C-terminal side. The remaining three-residue fragment (F(Sezn)A) is 18 Da lighter than expected for an unmodified peptide containing SeCys. Although the TruD-treated peptide gave extensive ion series to the N terminus, the PatD-treated peptide with multiple heterocycles could only be sequenced by combining multiple ion series (Figure S1 A and B).

We repeated the experiment by using a different PatE(Se) with the cassette sequence ITACITFC and observed (mass spectrometry) Se incorporation. After heterocyclisation with TruD, we treated the resulting peptide with protease to yield the peptide ITA(Sezn)ITF(Sezn)AYDGE, which was then macrocyclised with purified PatG^11^ in vitro, as previously described.^11^ The macrocycle has the expected mass for two Se atoms (Figure 2 C), and fragmentation analysis (Figure S2) confirmed the macrocycle structure. PatG will only macrocyclise a peptide with a heterocycle (or Pro) immediately N-terminal to the AYDGE motif.^12^ Taken together, these data establish that TruD and PatD are capable of heterocyclising SeCys.

The heterocyclisation reaction of SeCys by PatD or TruD was complete in 30 min. This is a similar time frame to the reaction of the natural substrate, thus indicating that SeCys is a suitable substrate for PatD/TruD. The reaction has been proposed to proceed through nucleophilic attack of the –XH on the adjacent amide to form a hemiorthoamide intermediate.^13^ This intermediate attacks the γ-phosphate of ATP to form a phosphorylated hemiorthoamide, which subsequently decomposes to thiazoline and inorganic phosphate. Se is considered to be more nucleophilic than S or O therefore it should be more reactive if hemiorthoamide formation is rate limiting. We lack a robust assay to detect changes in rate of less than an order of magnitude and suggest any effect is within this range for the SeCys variant.

Sezn compounds might have different biological as well as chemical properties from their oxygen and sulfur relatives. Their widespread testing and thus exploitation have been limited by the difficulty of producing them in the laboratory. An enzymatic route from commercially available SeCys is a relatively benign method for the production of Sezn-containing natural products. Such Sezn variants could be directly useful or form part of larger synthetic schemes. Furthermore, as many natural products contain thiazolines/thiazoles or oxazolines/ oxazoles and thus a heterocyclase is used during biosynthesis, one can envisage biosynthetic routes to Se variants of several natural products. Where the pathway can be reconstituted in *E. coli*, production of SeCys variants can be trivially accomplished by medium supplementation; this enables production by both ribosomal and nonribosomal peptide systems. Where microorganisms other than *E. coli* have to be used, either growth medium can be supplemented with SeCys (akin to *E. coli*) or SeCys-containing peptide substrate (made either synthetically or in *E. coli*) can be added to crude extract or purified proteins.

It has been reported that ∼25 % of sequenced prokaryotic genomes contain the genes necessary for selenocysteine biosynthesis.^14^ The demonstration that two heterocyclases can process SeCys raises the prospect of significant numbers of selenium-containing peptide-derived natural products to be discovered.

## Experimental Section

We constructed a modified PatE that has a single eight-residue (ITASITFC) product cassette and is flanked by the normal leader sequence and macrocyclisation tag. A C-terminal His-tag was added to the construct to aid purification. The construct was over expressed in *E. coli* in minimum medium supplemented with selenocysteine (100 mg L^−1^), as described by Salgado et al.^15^ The final yield of purified PatE(Se) was 22 mg per litre of bacterial culture. Analysis of the protein by mass spectrometry showed that the incorporation of SeCys ranged from 60 to 100 % (estimated by MALDI). Usually, we obtained ∼75 % incorporation, and the protein was a mixed PatE sample containing the SeCys-incorporated PatE(Se) (6891.4 Da) and cysteine (denoted PatE(S), 6844.7 Da), although on occasion we were able to obtain 100 % Se substitution. The expression conditions are described further in the Supporting Information.

PatE(Se) was treated with purified TruD (100 μM PatE, 5 μM TruD, 5 mM ATP, 5 mM MgCl_2_, 150 mM NaCl, 10 mM HEPES, pH 7.4, 1 mM tris-(2-carboxyethyl)phosphine hydrochloride (TCEP), 30 min at 37 °C) and both PatE(Se) and PatE(S) lose 18 Da; this is consistent with the dehydrative heterocyclisation of cysteine and selenocysteine (Figure 1). We incubated PatE(Se) and PatE(S) with iodoacetamide (IAA) and analysed the products by mass spectrometry (Figure 1). IAA forms a covalent adduct with free thiol or selenol, thereby adding 57 Da to the molecular weight (Figure 1 and Table S1). After treatment of PatE(Se) with TruD there is no change in mass upon addition of IAA (Figure 1), thus indicating that the free selenol has been consumed. We repeated the experiment with both PatE(Se) and PatE(S), but with PatD replacing TruD. Under the same experimental conditions, we observed losses of 36 and 54 Da (two and three water molecules, respectively) for both, thus indicating incomplete heterocyclisation (Figure 1). Addition of IAA after PatD incubation did not result in a mass shift; thus suggesting that the thiol of PatE(S) and selenol of PatE(Se) were completely heterocyclised. This would imply incomplete heterocyclisation of the alcohols by PatD.

Intact protein mass was determined by LC-ESIMS on a Waters 2795 HPLC and Waters LCT MS. Sample (20 μL, ca. 50 μM) was injected onto a Waters Massprep column (2.1×5.0 mm). The sample was washed with water/acetonitrile (ACN) with 1 % formic acid (95:5) for 5 min to desalt the protein and eluted with a gradient from 95:5 to 5:95 over 5 min. Then the column was returned to the initial conditions and reequilibrated for 10 min. Positive ionisation ESI-MS was carried out with scanning from *m*/*z* 500 to 2500 in 1 s at a capillary voltage of 3500 V and cone voltage of 30 V by using an RF lens of 500 Hz and Waters Masslynx v. 4.0 software. Multiply charged ion series were transformed to mass data by using the MaxEnt I deconvolution program over a range of 6000–8000 Da at a resolution of 0.1 Da with peak width at half height on the most intense peak in the charged ion series.

Peptides were separated by using a nanoLC Ultra 2D plus loading pump and nanoLC AS-2 autosampler equipped with a nanoflex cHiPLC chip-based chromatography system (Eskigent, Dublin, CA), loaded with a ChromXP C18-CL trap and column (Eskigent, Dublin, CA). The peptides were eluted with a gradient of increasing ACN concentration in water, containing 0.1 % formic acid (5–35 % ACN over 45 min, 35–50 % over a further 3 min, followed by 95 % ACN to clean the column, before re-equilibration with 5 % ACN). The eluents were then sprayed into an ABSciex 5600 TripleTOF instrument (ABSciex, Foster City, CA). Product ion scans of the target masses of 892.9, 901.9 and 910.9 Da were acquired with a TOF MS/MS range of 95–2000 Da with 0.5 s acquisition cycle time at unit resolution and also at open resolution to observe the Se isotope pattern. Ion spray voltage was set to 2500 V, declustering potential was set at 100 V, and collision energy was set at 65 V with a spread of 15 V. The spectra obtained were manually inspected by using PeakView 1.2 software (ABSciex).

## Supplementary material

As a service to our authors and readers, this journal provides supporting information supplied by the authors. Such materials are peer reviewed and may be re-organized for online delivery, but are not copy-edited or typeset. Technical support issues arising from supporting information (other than missing files) should be addressed to the authors.
