# Supplementary material for: An Enzymatic Route to Selenazolines
Source: Chembiochem. 2013 Feb 18;14(5):564–7. doi: 10.1002/cbic.201300037 (PMC3625746; doi:10.1002/cbic.201300037)
Supplement: Supplementary file 1 [file cbic0014-0564-SD1.pdf]

CHEMBIOCHEM

Supporting Information

© Copyright Wiley-VCH Verlag GmbH & Co. KGaA, 69451 Weinheim, 2013

An Enzymatic Route to Selenazolines

Jesko Koehnke,[a] Falk Morawitz,[a] Andrew F. Bent,[a] Wael E. Houssen,[b, c] Sally L. Shirran,[a] Matthew A. Fuszard,[a] Iain A. Smellie,[a] Catherine H. Botting,[a] Margaret C. M. Smith,[b] Marcel Jaspars,*[c] and James H. Naismith*[a]

cbic_201300037_sm_miscellaneous_information.pdf

Table S1 Compounds for which MS data is presented in Figure 1

Material	Change relative to native	Structure	Exp (Da)	Obs (Da)
PatE(Se)	Native	Leader- 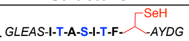	6892.6	6891.4
PatE(S)	Native (incomplete Se substitution)	Leader- 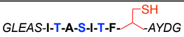	6845.6	6844.7
PatE(Se) + IAA	Addition to Se-H, + 57 Da	Leader- 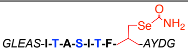	6949.6	6949.0
PatE(S) + IAA	Addition to S-H, + 57 Da	Leader- 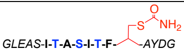	6902.6	6901.6
PatE(Se) + TruD	Loss of water, - 18 Da	Leader- 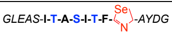	6874.6	6873.9
PatE(S) + TruD	Loss of water, - 18 Da	Leader- 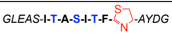	6827.6	6827.0
PatE(Se/S) + TruD + IAA	S and Se heterocycles do not react with IAA , therefore expected mass same as TruD addition, this is observed.			
PatE(Se) + PatD	Loss of up to four waters, -72, 54, 36,18 Da	Leader- 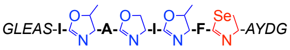 (or various arrangements of 3, 2 or 1 heterocycles)	6820.6/6838.6/6856.6/6874.6	N.O./6838.4/6856.0/N.O
PatE(S) + PatD	Loss of up to four waters, -72, 54, 36,18 Da	Leader- 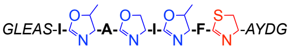 (or various arrangements of 3, 2 or 1 heterocycles)	6773.6/6791.6/6809.6/6827.6	N.O./6792.4/6809.7/N.O.
PatE(Se/S) + PatD + IAA	If Se and S heterocycles are formed they do not react with IAA, therefore same as PatD addition.<br>If Se and S heterocycles are not formed (i.e. oxazolines formed only), then would expect + 57Da.<br>No change observed, we thus conclude all selenol (and thiol) heterocyclized, incomplete reaction due to unreacted alcohol.			
PatE leader sequence	MNKKNILPQQGQPVIRLTAGQLSSQLAELSEEALGDA			

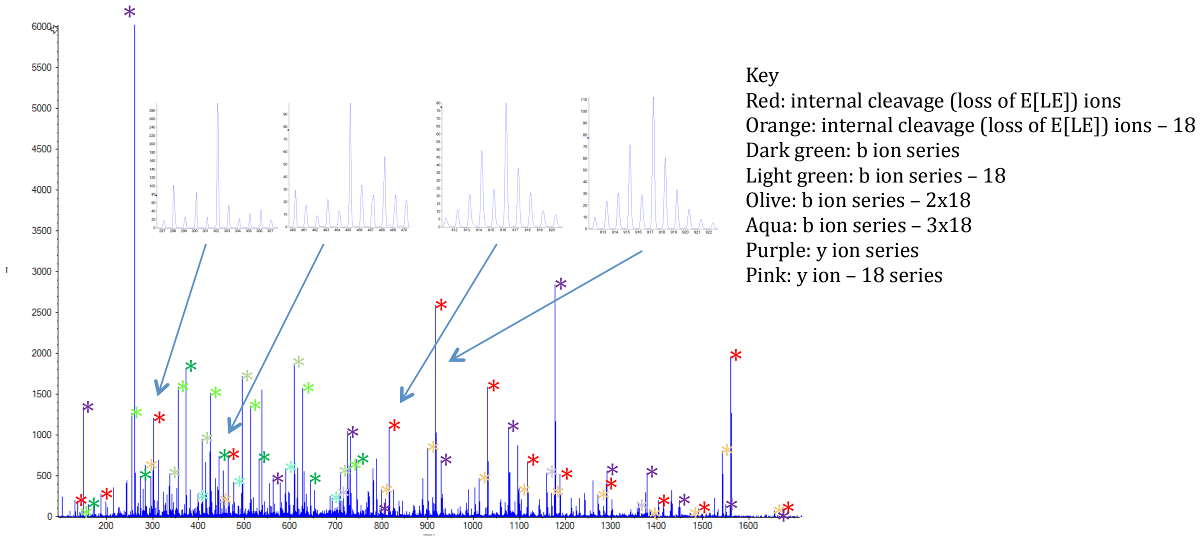

Figure S1A: MS/MS fragmentation of GluC derived PatE(Se) peptide after treatment with TruD. The internal cleavage ion series (red) allowed us to sequence the proteolytic fragment from the N- to C-terminus with the exception of the residues immediately before and after the heterocycle. This three-residue fragment is 18 Da lighter than would expected for unheterocyclized SeCys. A full list of identified peaks can be found in Table S2. The characteristic isotope pattern for Se appears as expected in the internal cleavage pattern where the heterocycle is present (masses 816.2103 and 917.2592 Da; both inset on right hand side). The pattern is absent where the heterocycle is not in the fragment (two examples shown inset on left hand side).

Table S2 Observed peaks in MS/MS fragmentation of GluC derived PatE(Se) peptide (S1A) after treatment with TruD and their origin.

	Int cleav (-LE)	Int cleav (-E)	Int cleav (-LE) -18	Int cleav (-E) -18	b ion	b ion - 18	b ion - 2x18	b ion - 3x18	y ion	y ion -18
A	1560.6152	1673.6967	1542.6029	1655.6817						
S	1489.5741		1471.5613		159.0768	141.0635				
I	1402.5439		1384.5321		272.1611	254.1496			1662.6810	
T	1289.4624		1271.4524		373.2085	355.1983	337.1875		1549.6013	
A	1188.4126		1170.4024		444.2459	426.2352	408.2243	390.1892	1448.5451	
S	1117.3757		1099.3639		531.2779	513.2666	495.2569	477.2463	1377.5138	1359.4967
I	1030.3432		1012.3326		644.3622	626.3509	608.3407	590.3293	1290.4720	
T	917.2592		899.2487		745.4086	727.3988	709.3881	691.3768	1177.3968	1159.3844
F	816.2103		798.2000						1076.3488	
het(SeCys)									929.2949	
A									796.3376	
Y	465.1623		447.1515						725.2989	707.2856
D	302.0991		284.1611						562.2366	
G	187.0707									
E	130.0492									
L									261.1447	
E									148.0598	

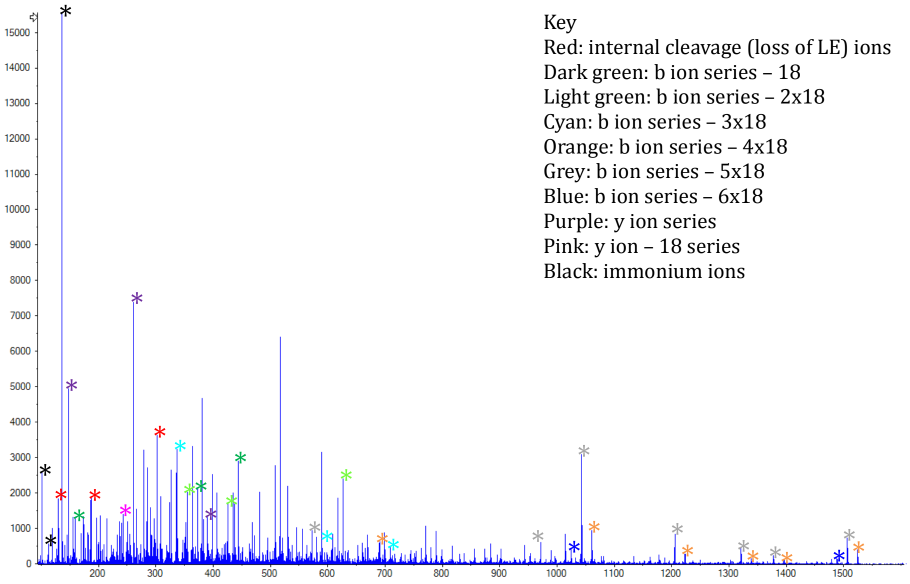

Figure S1B: Observed peaks in MS/MS fragmentation of GluC derived PatE(Se) fragment after treatment with PatD. The spectrum is closely related to the spectrum shown in Figure S1A produced by TruD. The additional heterocycles induce further fragmentation increasing the complexity of the spectrum. The internal cleavage (red) and b-ion -5 x H_2O (grey) series allowed us to sequence through the selenazoline heterocycle. A full list of identified peaks can be found in Table S3.

Table S3 Observed peaks in MS/MS fragmentation of GluC derived PatE(Se) peptide after treatment with PatD and their origin.

	Int cleav (-LE)	b ion - 18	b ion - 2x18	b ion - 3x18	b ion - 4x18	b ion - 5x18	b ion - 6x18	y ion	y ion - 18	Imm
A										
S		159.0776								
I										
*hetT		373.2098	355.1994	337.1889						
A		444.2471	426.2352							
*hetS										
I			626.3530	608.3421		572.1792				
*hetT				709.3899	691.3796					
F										120.0817
het(SeCys)						971.3917				
A					1060.4400	1042.4294	1024.4185			
Y					1223.5042	1205.4938				136.0767
D	302.1000				1338.5356	1320.5205				
G	187.0728				1395.5506	1377.5402				
E	130.0509				1524.5923	1506.5841	1488.5765	390.0734		102.0559
L								261.1458	243.1349	
E								148.0613		102.0559

* two of the three hetT/S/T sites are heterocyclised but it is not possible to determine which since the S and T side-chains also loose 18 Da in CID.

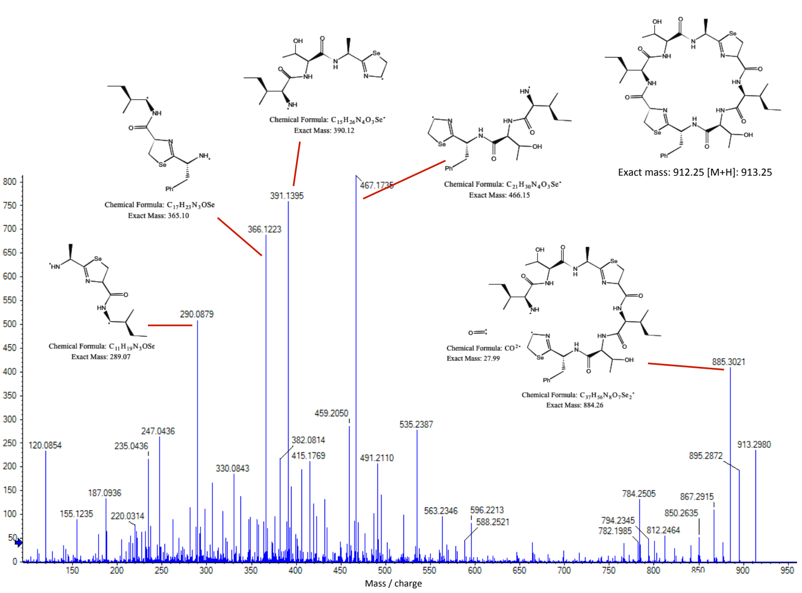

Figure S2: MS/MS fragmentation of the enzymatically produced peptide macrocycle containing two Sezn rings. The unusual fragmentation pattern of the macrocyclised peptide is hypothesized to originate with the cleaving and excision of the carbonyl group adjacent to either/both of the heterocycles. This results in the complex fragmentation spectrum characteristic of macrocycles.

Additional Methods

Cloning, expression and purification of PatE pre-pro-peptides

PatE constructs were cloned into the pBMS vector (a gift from H. Liu) with a C-terminal His_6-tag and expressed in *Escherichia coli* BL21 (DE3) cells as modified from the Salgado *et al*. procedure. 30 ml of the PatE expressing strain were grown overnight at 30 °C in Luria-Bertani medium, spun down and washed three times with 40 ml minimal media. The washed cells were used to inoculate 1 L of minimal media supplemented with 5 % glycerol and 1.1 g glucose-free nutrition mix (Molecular Dimension). The culture was grown at 30 °C until an optical density at 600 nm of 0.6 when amino acids were added (See Table S4). Fifteen minutes after amino acid addition, 1 mM isopropyl β-D-1-thiogalactopyranoside (IPTG) and 100 mg selenocystine (Sigma Aldrich) were added and the culture incubated at 30 °C overnight. Cells were harvested by centrifugation at 4,000 x g for 15 min at 20 °C, re-suspended in urea lysis buffer (8 M urea, 150 mM NaCl, 20 mM Tris pH 8.0, 20 mM Imidazole and 3 mM b-mercaptoethanol (BME) and lysed by sonication at 15 microns for 4 min (SoniPrep 150, MSE). The lysate was cleared by centrifugation at 40,000 x g, 20 °C for 45 min followed by passage through a 0.45 µm filter. The cleared lysate was applied to a Ni-sepharose FF column (GE Healthcare) prewashed with urea lysis buffer and protein eluted with 250 mM imidazole. The protein was then dialysed into 150 mM NaCl, 20 mM HEPES pH 7.4, 1 mM TCEP in six steps of decreasing urea concentration to induce refolding and then subjected to size-exclusion chromatography (Superdex 75, GE Healthcare) in the same buffer. Peak fractions were pooled and concentrated to 1 mM.

Table S4 - Amino acid mixture for seleno-cysteine growth per 1 L culture.

1.6 g	Serine
1 g	Leucine
0.4 g	Alanine, gluatmate, glutamine, arginine, glycine
0.25 g	Aspartate
0.1 g	Lysine, threonine, phenylalanine, asparagine, histidine, proline, tryrosine, tryptophan, methionine
0.05 g	Isoleucine, valine

Cloning, expression and purification of enzymes

Codon-optimized full length PatD and TruD were cloned into the pJexpress 411 plasmid (DNA2.0 Inc., USA) with an N-terminal His_6-tag, with TruD containing an additional Tobacco Etch Virus (TEV) protease cleavage site. Both enzymes were expressed in *Escherichia coli* BL21 (DE3) cells

grown on auto induction medium for 48 h at 20 °C. Cells were harvested by centrifugation at 4,000 x g, 4 °C for 15 min.

PatD and TruD were re-suspended in 500 mM NaCl, 20 mM Tris pH 8.0, 20 mM Imidazole and 3 mM BME and supplemented with 0.4 mg DNAse g^{-1} wet cells (Sigma) and complete protease inhibitor tablets (EDTA-free, Roche). Cells were lyzed by passage through a cell disruptor at 30 kPSI (Constant Systems Ltd.) and the lysates cleared by centrifugation at 40,000 x g, 4 °C for 45 min. Cleared lysates were applied to a Ni-sepharose FF column (GE Healthcare) pre-washed with lysis buffer and the protein eluted with 250 mM Imidazole.

TruD was passed over a desalting column (Desalt 16/10, GE Healthcare) in 100 mM NaCl, 20 mM Tris pH 8.0, 20 mM Imidazole 3 mM BME. Tobacco Etch Virus (TEV) protease was added to the protein at a mass-to-mass ratio of 1:10 and the protein digested for 1 h at 20 °C to remove the His_6-tag. Digested protein was passed over a second Ni-sepharose column with the flow-through concentrated prior to size-exclusion chromatography. PatD was applied to size-exclusion chromatography directly from first Ni-sepharose elute.

Both enzymes were loaded on to a Superdex 200 gel filtration column (GE Healthcare), pre-equilibrated and run in 150 mM NaCl, 10 mM HEPES pH 7.4, 1 mM TCEP. Peak fractions were pooled and the proteins concentrated to 100 µM for use in *in vitro* reactions.
